# Hump-Shaped Density-Dependent Regulation of Mosquito Oviposition Site-Selection by Conspecific Immature Stages: Theory, Field Test with *Aedes albopictus,* and a Meta-Analysis

**DOI:** 10.1371/journal.pone.0092658

**Published:** 2014-03-28

**Authors:** Gideon Wasserberg, Nicholas Bailes, Christopher Davis, Kim Yeoman

**Affiliations:** 1 Biology Department, University of North Carolina at Greensboro, Greensboro, North Carolina, United States of America; 2 Institute for Global Interdisciplinary Studies, Villanova University, Villanova, Pennsylvania, United States of America; 3 Biology Department, Messiah University, Mechanicsburg, Pennsylvania, United States of America; Fundação Oswaldo Cruz, Brazil

## Abstract

Oviposition site selection by gravid females is an important determinant of the distribution, abundance, and dynamics of dipteran hematophagous insects. The presence of conspecific immature stages in a potential oviposition site could, on the one hand, indicate the suitability of that site but on the other hand could indicate the potential for intraspecific competition. In this paper, we present a graphic model suggesting that the trade-off between these two opposing forces could result in a hump-shaped density-dependent relationship between oviposition rate and conspecific immature stage density (hereafter, the “Hump-shaped regulation model”) with positive effects of aggregation prevailing at low densities and negative effect of intraspecific competition prevailing at higher densities. We field-tested the predictions of this model at both the egg- and the larval levels with *Aedes albopictus* and evaluated if and how these relationships are affected by resource enrichment. We found support for the hump-shaped regulation model at both the larval and the egg levels. Using oviposition cups containing varying numbers of conspecific larvae, we showed that the oviposition activity of *Ae. albopictus* first increases and then decreases with larvae number. Medium enrichment resulted in higher hatching rate, and demonstrated linear relations for the no-enrichment treatment where larvae density range was low and hump-shaped relationship for the enriched medium that had a wider larvae density range. Using pairs of oviposition cups, we showed that at low egg densities mosquitoes laid more eggs on substrates containing pre-existing eggs. However, at higher egg densities, mosquitoes laid more eggs on a virgin substrate. Based on our results and on a meta-analysis, we suggest that due to study design or methodological shortcomings the hump-shaped regulation model is often left undetected and that it is likely to be more common than currently thought.

## Introduction

For organisms lacking parental care and where larval dispersal is limited, oviposition-site selection decisions by gravid females are critical fitness-enhancing choices with critical implications to the distribution, abundance and dynamics of those populations [1]–[9]. This situation could apply to many vernal-pool and container inhabiting organisms. With most such organisms, it has been demonstrated that predation risk, abundance of food resources, and the presence of conspecifics are important factors affecting this decision with gravid females typically avoiding sites with predators and attracted to sites with indication of abundant food for their offspring [6], [10]–[17]. Regulation of the oviposition behavior of mosquitoes and other bloodsucking insects is an issue that is studied intensively due to its implications for population dynamics, evolutionary trajectories, and pest and disease control [1], [4], [10], [11], [18]–[25].

**Figure 1:**
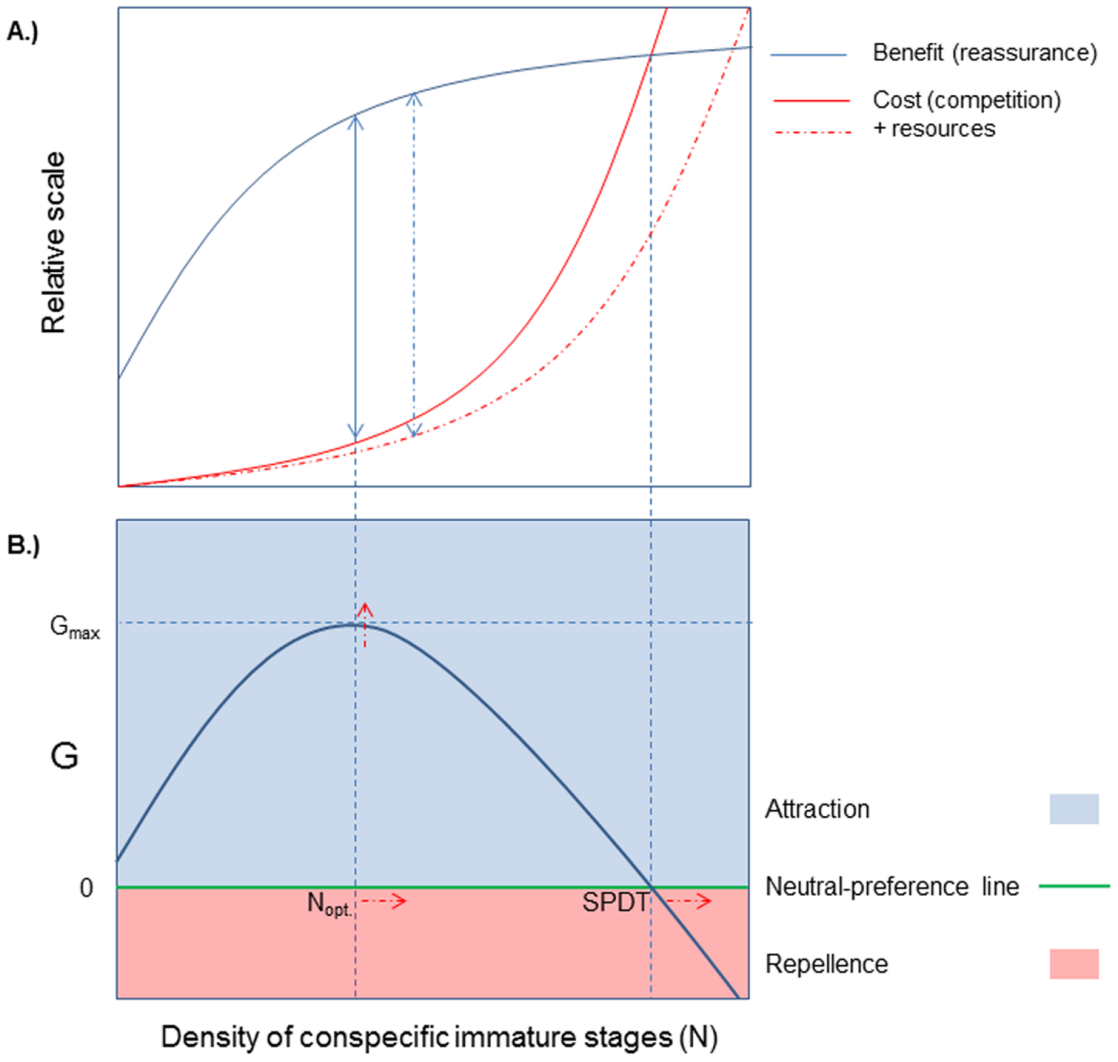
The Hump-Shaped Regulation (HSR) model. (A) Cost-benefit model of the relationship between conspecific immature stage density and the reassurance benefit due to egg or larvae aggregation and cost of competition in the absence and presence of resources. Two-headed smoothed and dashed arrows represent the maximal net benefit in the absence and presence of resources, respectively. (B) The trade-off between the benefit of reassurance and the cost of intra-specific competition should result in a hump-shaped relationship between conspecific immature density and fitness (G). G_max_ indicates the maximal fitness and N_opt_ indicates the density at which it occurs. The switching preference density threshold (SPDT) indicates the conspecific density at which this fitness line crosses the neutral-preference line. This neutral-preference line dissects the state-space into regions of conspecific attraction (G > 0, light blue) and repellence (G < 0, pink). Hence, at densities below the SPDT attraction to conspecific immature stages should be exhibited while above it repellence from conspecific immature stages should be exhibited. Red dashed arrows indicate the expected shift in N_opt_, G_max_, and SPDT due to resource addition.

The effect of conspecific immature stages on the oviposition site-selection of gravid mosquitoes has received a lot of attention, however, the results are highly conflicting with some studies reporting no effect [24], [26]–[32], some reporting positive effects [27]–[29], [32]–[47], some reporting negative effects [31], [33], [35], [36], [48]–[55], and some reporting mixed effects [27], [33], [36], [55]–[60]. In these studies, most attention has been given to the identification of oviposition attractants and repellants but no serious attempt has been given to try to develop a unifying theory that would explain these conflicting observations. Of particular interest are studies reporting a density-dependent shift in the effect of immature stages on oviposition response from positive effect at low densities to negative effect at higher densities [27], [36], [55]–[60]. Such a pattern could be explained as an outcome of the interaction between an Allee effect (positive relationship between the fitness [or a component thereof] of an individual and density of conspecifics [61]) and intraspecific competition. This interaction should result in a hump-shaped relationship between conspecific immature density and oviposition rate termed, hereafter, the “hump-shaped regulation (HSR) model” (Fig. 1). According to this model, female mosquitoes seeking oviposition sites face the challenge of finding suitable site. Further, we assume that natural selection should mold mosquito oviposition site selection behavior in a manner that would maximize its fitness (G) [5], [7], [23], [34]. Considering only the effect of conspecific immature stages (eggs, larvae), the presence of these in a site could indicate the suitability of that site in terms of, among others, food for their larvae, site persistence, lack of predators, and appropriate a-biotic conditions [5], [34], [56], [59]. We refer to this fitness benefit as the “reassurance effect” (R). On the other hand, the presence conspecific immature stages could inflict a fitness cost in terms of larval competition (C) [5], [7], [33], [62]–[64]. Both effects are communicated via specific chemical cues [1], [65], [66]. Fitness is, therefore, the difference between the benefit of reassurance and the cost of intraspecific competition (G = R – C) (Fig. 1A). Both R and C are a function of conspecific immature stage density (N). Due to a biological limit on oviposition capacity and the density-dependent nature of competition we expect R and C to increase asymptotically and exponentially with N, respectively (Fig. 1A). This would result in an asymmetric hump-shaped fitness function with peak fitness (G_max_) occurring at some intermediate level of conspecific immature stage density, which we term ‘optimal density’ (N_opt_) (Fig. 1B) and a Y-axis intercept at a certain low but positive fitness value. The X-axis intercept corresponds to the density at which the benefit of reassurance equals the cost of competition (R = C) (Fig. 1A). At this point G = 0 (Fig. 1B). At lower densities G > 0 while at higher densities G < 0. We term this X-axis intercept as the ’Switching Preference Density Threshold’ (SPDT) because below it oviposition sites containing conspecifics should provide a positive fitness reward and be perceived as attractive while at densities above it fitness reward would be negative and perceived as repulsive (Fig. 1B). At the SPDT, a neutral response is expected. This hump-shaped fitness curve is expected to be reflected by mosquito’s oviposition rates: at lower range of conspecific densities oviposition rate should increase with density, at an intermediate density range oviposition rate should decrease with density, at a higher density range neutrality should be exhibited, while at high densities repellence should be exhibited with gravid females steering away from sites containing conspecifics.

Although, to date, only five studies involving mosquitoes have described such a hump-shaped relationship [36], [55], [56], [59], [60], we believe that this pattern is likely to be the norm rather than the exception. Specifically, we suggest that the dearth of observations of this hump-shaped relationship pattern and the schizophrenic nature of observations in this field are likely to be the outcome of the design of previous studies that tended to explore only a limited range of conspecific densities. Hence, in contrast to most previous studies, here we studied the effects of conspecific immature stage densities on the oviposition response of gravid mosquitoes over a wide range of densities at both the egg and the larval stages. Comparison of the oviposition response to two different immature stages in the same study system should provide insights regarding the ability of gravid females to detect and respond to cues indicating potential future- (conspecific eggs) and current larval competition [33]. In addition, we attempted to evaluate whether resource addition affects these responses. We hypothesized that enrichment of rearing-medium would mitigate the competitive effect resulting in a shift of N_opt_ and SPDT to higher conspecific densities and increase in overall oviposition rate (Fig. 1B). Finally, we conducted a meta-analysis of published data to evaluate the generality of this HSR model among mosquitoes.

## Materials and Methods

### Study area and study design

**Figure 2:**
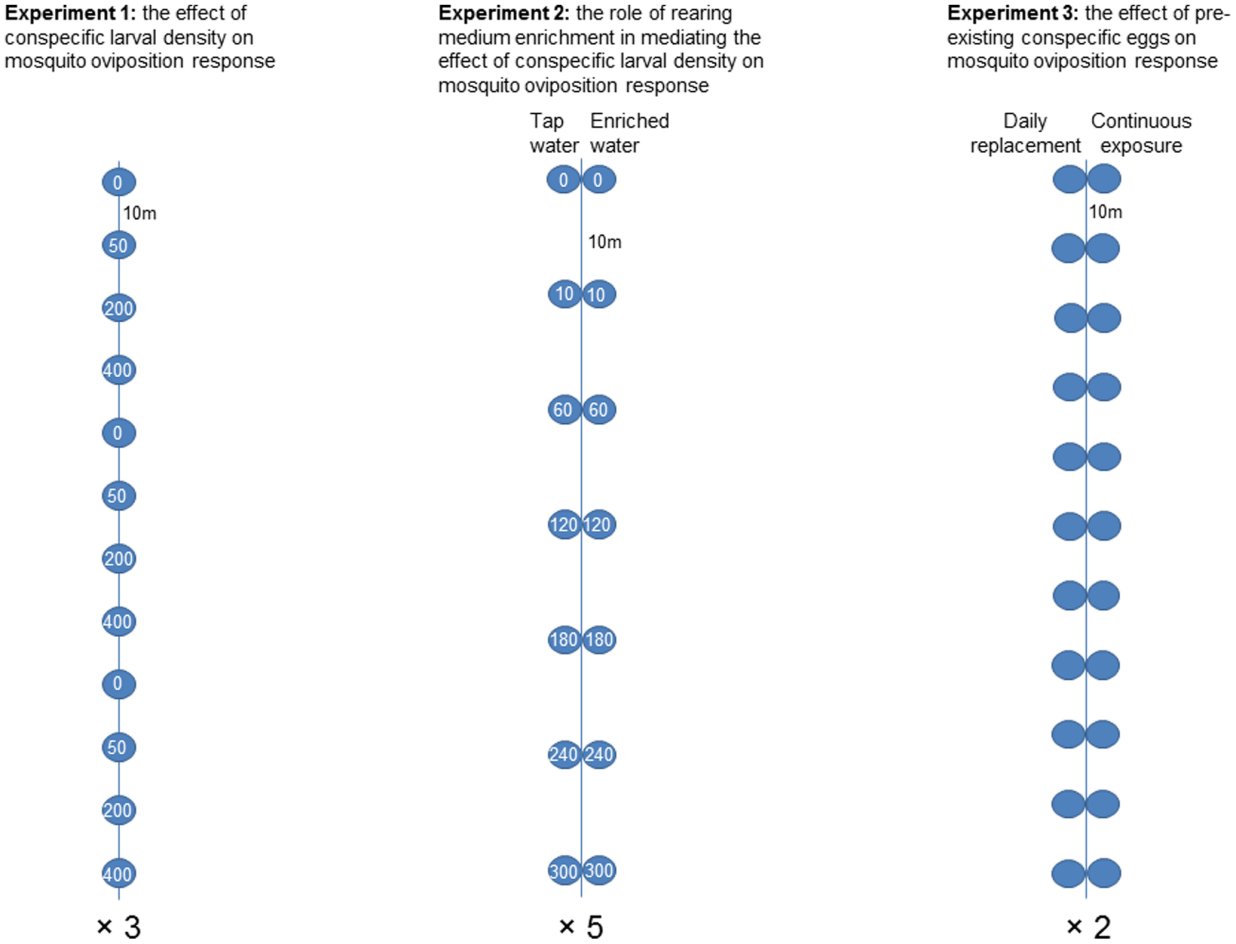
Experimental design. Transect are represented by lines and blue circles represent the oviposition cups. Numbers inside the circles represent the initial number of larvae introduced (experiments 1 and 2). Numbers underneath transects represent the number of spatial replicates.

Peabody Park is a thirty-four acre recreational and research deciduous forest on the northern side of the University of North Carolina at Greensboro campus. Its average elevation is 241 meters above sea level and soil texture is loamy. A system of several creeks, part of the Haw River Basin, flows throughout the park. In this park the predominant container breeding mosquito is *Aedes albopictus* (Say) [17]. This study comprised three experiments. The conspecific eggs experiment took place between July 7th and August 25th 2011, the medium enrichment experiment took place between September 7–14, 2011, and the conspecific larval experiment took place August 20^th^ – September 20^th^, 2012. All experiments were conducted using oviposition cups distributed, at 10 m intervals, along straight transects running through the forest with distance between transects ≥ 20 m (Fig. 2). Oviposition cups were black plastic cups (14.3 cm height, 6.5 and 9 cm diameter bottom and top, respectively) filled two-thirds (370 ml) with dechlorinated tap water and containing a rolled germination paper as an oviposition substrate (ovistrip) (25.5×9.5 cm, Anchor Paper Company, St. Paul, Minneapolis, USA) that was secured to cup’s lip with a black binder clip. Cups were punctured 5cm below the lip to prevent overflow due to rain storms. Following specified days of exposure (differing among experiments) ovistrips were collected and eggs were counted in the lab using a dissecting scope (Olympus SZ63, X4). Larvae used for the experiments were produced from local egg collections and supplemented, as needed, by *Aedes albopictus* eggs from Charles Apperson’s lab colony at North Carolina State University. A sample of field collections (100 eggs per transect taken randomly from several stations along each transect) was reared to adulthood to confirm species identity. All emerging adults were *Aedes albopictus*.

The effect of conspecific larvae Two experiments were conducted. One tested the effect of larval density on mosquito oviposition response and the second tested the effect medium enrichment on this relationship. In experiment 1 (Fig. 2, left), consisting of five replicate sessions, we used three transects (>100 m apart) containing 12 stations spaced 10 m apart. In each transect we stocked cups with three replicates of four levels of lab reared 1^st^ -2^nd^ instar *Ae. albopictus* larvae: 0, 50, 200, and 400. These numbers correspond to larval densities where increasing, null, and decreasing trends were previously reported for *Ae. aegypti* [56], [60]. At each sampling session, larval level treatments were distributed randomly among stations of each transect. Ovistrips were left exposed for five days (less than the time it took for 1^st^/2^nd^ larvae to complete metamorphosis) and then collected for egg counting in the lab. At each collection, larvae and cup-water were also collected and transferred in a large (1242 mL) nylon bag (Whirlpack, Nasco, Fort Atkinson, WI) to the lab where larvae and pupae were counted. Cups were replenished with fresh dechlorinated water and a new ovistrip was inserted to each cup.

In experiment 2 (Fig. 2, center) (1 session), we used five transects containing 7 stations each (10 m between stations, ca. 30 m between transects). Each station consisted of a pair of oviposition cups. One cup was stocked with regular dechlorinated tap water and the other with a 1-week old leaf infusion composed of a 1 kg mixture of ca. 1∶1 Hickory spp. and White Oak senescent leaves in a 20-liter container (hereafter, medium enrichment treatment) (following, [17]). In each station, cup-pairs received the same number of *Ae. albopictus* eggs. We used 7 levels of egg numbers: 0, 10, 60, 120, 180, 240, and 300; with their location randomized within each transect. Eggs were given 2 days to hatch in the cups and then ovistrips were introduced. Oviposition strips and cup-water were collected 5 days later and larvae in the cup-water and eggs on the ovistrips counted in the lab.

The effect of conspecific eggs In experiment 3 (Fig. 2, right), we tested during eight sampling sessions, the effect of pre-existing conspecific eggs on subsequent oviposition by gravid mosquitoes. We applied a paired-cup design using two 11-stations transects containing a pair of oviposition cups in each station (10 m between stations, ca. 100 m between transects). In one cup of each pair ovistrips were replaced daily (daily-replacement cups) and in the other ovistrips were left exposed (continuous-exposure cups) for varying number of days (1, 2, 3, 4, 6, 8, and 10 days). Given this arrangement, oviposition-site-seeking gravid mosquitoes arriving at a station are faced with a choice to either oviposit on a conspecific-eggs free ovistrip (the daily-replacement cup) or on an ovistrip containing pre-existing conspecific eggs (the continuous-exposure cup), with the number of pre-existing eggs expected to increase with exposure time. Sampling sessions differed with respect to exposure times with all cups within each session exposed for the same number of days. Exposure times were assigned randomly among the different sampling sessions. Except for exposure time of 1-day which received two replicate sessions, all other exposure times received a single replicate session. The cumulative number of eggs in the daily-replacement treatment was calculated as the sum of the daily egg counts for the entire duration of the prescribed exposure. For the continuous-exposure treatment, the cumulative number of eggs was the final egg count counted once at the end of the prescribed exposure time.

**Figure 3:**
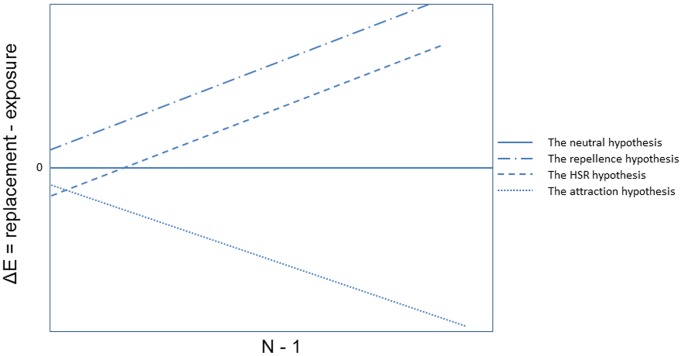
Predictions of the four competing hypotheses in terms of the difference in the cumulative number of eggs between the daily-replacement and the continuous-exposure cups at each station with respect to the cumulative number of eggs in the daily-replacement cups.

If the presence of pre-existing conspecific eggs does not affect oviposition behavior, then there should be no difference between the cumulative number of eggs in the daily-replacement- and the continuous-exposure cups throughout egg density range (the neutral hypothesis) (Fig. 3). If gravid females avoid substrates where conspecific eggs have previously been laid (the repellence hypothesis) than we expect that the daily**-**replacement cups would have more eggs throughout the egg range and this preference should increase with the number of eggs laid (Fig. 3). If gravid females are attracted to substrates where conspecific eggs have previously been laid (the attraction hypothesis) than we would expect that the daily**-**replacement cups would have less eggs than the continuous-replacement cups throughout the egg range. This preference for the continuous-exposure cups should increase with the number of pre-existing eggs (Fig. 3). Finally, if conspecific eggs are attractive at low densities but repellant at high densities (the HSR hypothesis) (Fig. 1), then, at low egg number the continuous-exposure cups should have more eggs but at higher pre-existing egg numbers the daily-replacement cups should have more eggs with this difference increasing with number of pre-existing eggs. Plotting these alternative hypotheses as the difference between the cumulative number of eggs in the daily-replacement and the number of eggs in the continuous exposure cups at each station (hereafter, ΔE) against the cumulative number of eggs in the daily replacement cups minus 1 (subtracting by 1 enables consideration of the outcomes only for ovistrips where at least one egg has been laid) enables simple distinction between the predictions of each model (Fig. 3). The neutrality hypothesis would predict a horizontal line with a 0 intercept, the repellence hypothesis would predict a line with a positive intercept and a positive slope, the attraction hypothesis would predict a line with a negative intercept and a negative slope, and the HSR hypothesis would predict a line with a negative intercept but a positive slope (Fig. 3).

To confirm lack of confounding effect due to age of the ovistrip (in the daily replacement cups ovistrips are fresh whereas in the continuous exposure they age with exposure time) we placed ovistrips in water-filled oviposition cups and aged them under simulated field conditions in an environmental chamber (27°C, 80%RH, 12∶12 hr. photoperiod) for 0, 2, 5, and 10 days. Then, along two 100 m long transects, we deployed 20 pairs of oviposition cups: one cup with the aged germination paper and the other with a fresh germination paper. Ovistrips were collected five days later and eggs were counted in the lab.

### Meta-analysis

**Figure 4:**
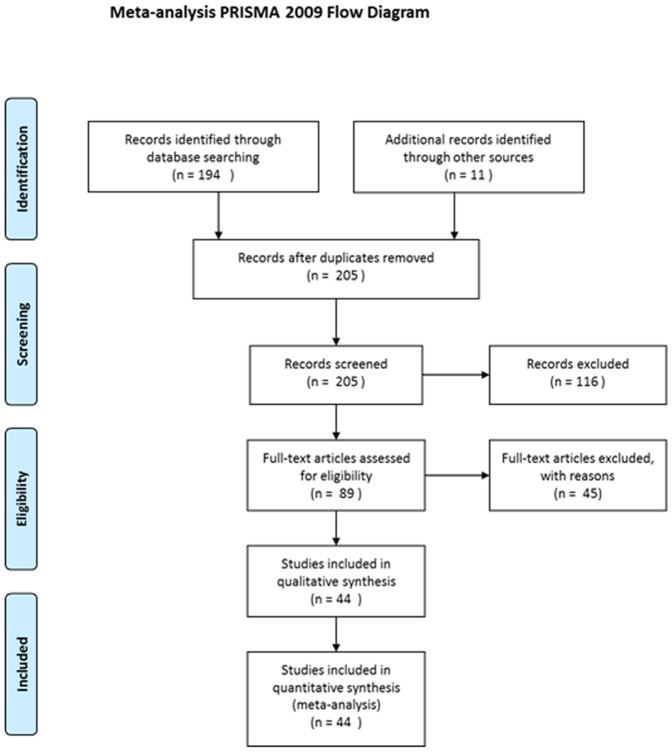
Meta-analysis flow diagram.

Literature search was conducted using ISI-Web of knowledge for all years using search code: Topic = (mosquito* and (oviposition or egg laying) and (conspecific or habitat selection or competition)). A total of 194 papers were found and an additional 11 were added based on relevant references mentioned in the reference list of relevant papers. Only papers considering the effect of conspecific eggs, larvae, or pupae on oviposition response of mosquitoes were included in the analysis (Fig. 4) resulting in a total of 44 papers that were used for the analysis. Studies were stratified by immature stage (eggs, larvae, pupae) and study type (Laboratory or field). We noted and placed these articles according to their finding regarding the effect of immature stage density on oviposition rate. For each paper we tried to estimate immature stage density based on the information provided in their ‘Methods’ section (See, Table S1).

### Data reduction and statistical analysis

Due to the nature of the data (count) and its high degree of overdispersion, we analyzed it using negative-binomial (NB) generalized-linear models [67]. NB models were preferred over Poisson models due to their superior fit to the data. Comparing AIC of the saturated models of experiments 1 and 2 (Tables 1, 2, respectively) between the NB- and the Poisson-regression models we found the NB models to fit substantially better in both cases (ΔAIC = 968.9 and ΔAIC = 1643.5, respectively). We tested for negative second-order polynomial relationship between final larval number in the cup and number of new eggs laid after controlling for the effect of transect and/or date (dummy variables). In both larval experiments, we used final larvae number per cup as the predictor variable since there was often a large reduction in the number of larvae from the number initially deployed. For the conspecific eggs experiment, NB model also best fitted the data (ΔAIC > 136 compared with a Gaussian and Poisson models) and therefore used for a Deviance analysis of that data. In all experiments involving a paired-cup design, we used a paired t-test to test for statistical difference. For the analysis of the ΔE data we used a simple linear regression analysis. For the meta-analysis we used contingency-table tests, goodness-of-fit tests, and proportion tests.

**Table 1:** Negative-binomial multiple-regression analysis of the relationship between the final number of larvae per container and number of *Ae. albopictus* eggs laid.

Variable	Coefficient	SE	z-value	P-value
Intercept	4.212	0.211	19.791	**<0.0001**
Larvae	5.414E-3	2.295E-3	2.359	**0.0183**
Larvae^2^	-1.563E-5	7.759E-6	–2.015	**0.0439**
Transect B	-9.95E-1	0.260	–3.818	**0.0001**
Transect C	-8.64E-2	1.993E-01	–0.434	0.6656
Date 9/20	-9.719E-01	2.590E-01	3.752	**0.0002**
Date 8/25	-4.296E-01	2.825E-01	–1.521	0.1283
Date 9/27	4.869E-01	3.892E-01	1.251	0.2109
Date 8/30	-7.757E-02	2.570E-01	0.302	0.7627
Date 9/6	-1.452E-02	2.620E-01	–0.055	0.9558

‘Transect’ and ‘Date’ are dummy variables controlling for spatial location and sampling date, respectively. ‘Larvae’ and ‘Larvae^2^‘ indicate 1^st^- and 2^nd^-order polynomial terms, respectively. Overdispersion parameter  =  2.66 (n = 85).

AIC: 841.1

**Table 2:** Negative Binomial Analysis of Deviance table for the effects of conspecific larvae number, medium enrichment, their interaction, and location (Transect) on the cumulative number of eggs laid by *Aedes albopictus* mosquitoes in oviposition cups.

Variable	DF	Deviance	Res. DF	Res. Dev.	P value
NULL			61	136.69	
Larvae	1	40.56	60	96.13	**< 0.0001**
Enrichment	1	16.61	59	79.52	**< 0.0001**
Transect	4	5.45	55	74.07	0.244
Larvae x Enrichment	1	10.89	54	63.18	**0.0009**

## Results

### Experiment 1: the effect of larval density

**Figure 5:**
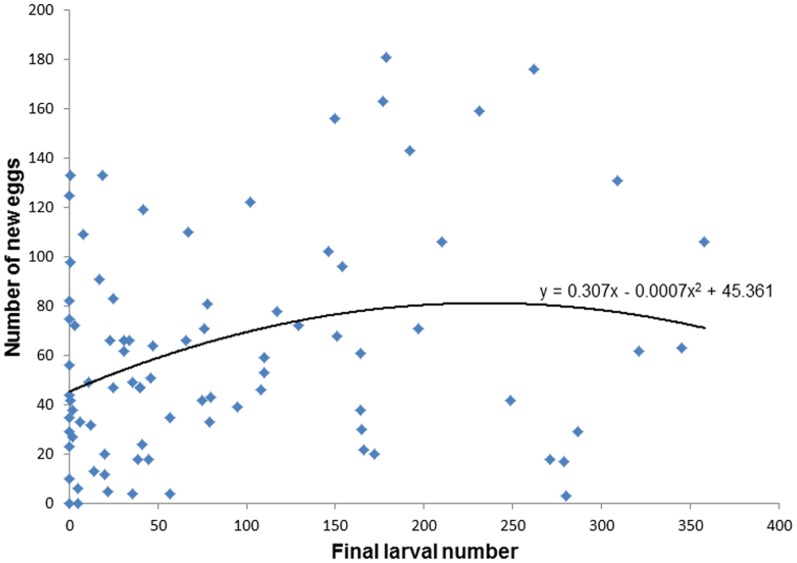
The effect of conspecific larvae number (measured as number of larvae in the cups by the end of the experiment) on *Aedes albopictus* oviposition response measured as number of eggs laid on an oviposition strip following 5 days of exposure. Least-squares 2^nd^-order polynomial regression plot of this relationship is presented.

Despite high variability in these data, after controlling for the effect of transect location and session date, the relationship between number of larvae and number of new eggs laid was consistent with a negative second-order polynomial relationship (Table 1), with number of eggs initially increasing, peaking at about 221 larvae (estimated by calculating the 1^st^-derivative of the regression line), and then gradually decreasing (Fig. 5). This model (Table 1) had a better fit compared to a model lacking the second-order polynomial larvae term (ΔAIC = 2.06) and compared with a model lacking both larval terms (ΔAIC = 1.78). The increasing trend at the lower range of larval densities is quite apparent up until approximately 130 larvae. Highest egg deposition occurred at a range between 179 and 262 larvae and thereafter number of egg appeared to be decreasing with larval density (Fig. 5). The descending part of this trend is highly variable yet statistically significant (Table 1). The general pattern exhibited here is consistent with the HSR model’s predictions (Fig. 1). We estimated the SPDT by calculating the larval number at which egg deposition would equal the intercept of the second-order polynomial regression line (Fig. 5) (representing expected egg deposition in the absence of conspecific larvae) and found it to be 438.55 larvae.

### Experiment 2: the effect of medium enrichment on the relationship between larval density and oviposition

**Figure 6:**
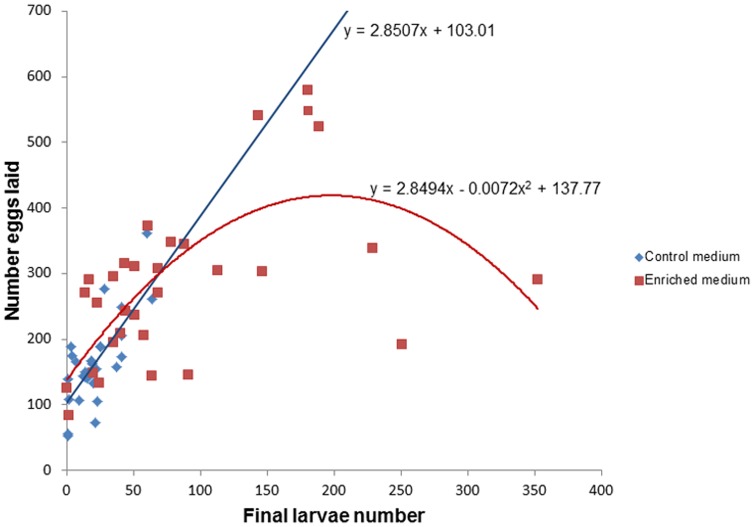
The rearing medium enrichment experiment. Least-squares regression plot of the relationship between conspecific larvae number of number of mosquito eggs laid per cup for enriched (red squares) and the water (blue diamond) media.

Conspecific larva number had a, general, positive effect on egg deposition (Table 2, Fig. 6). As expected, rearing medium enrichment increased egg deposition by 81% from a mean of 162.4 to 293.9 eggs per cup (Table 2, Fig. 6). However, the effect of larval number differed between the treatments (Table 2). A highly significant difference in eggs hatching rate was observed between the enriched and the control water media (60.1 and 13.4 percent, respectively) (Pearson’s Chi-squared test: χ^2^ = 2129.5, P<0.0001). This resulted in larval number range being substantially lower in the control medium (range: 4–41, mean: 14.65 larvae/cup) compared with the enriched medium (range: 0–352, mean 91.96 larvae/cup). Hence, since larval number in the control and the treatment cups differed, we could not evaluate our original hypothesis. Nonetheless, we tested the HSR model predictions by fitting a second-order polynomial NB regression for data of each treatment. For the control medium, only the linear term (positive slope) was significant (eggs  =  4.735+0.015Larvae, z = 4.653, P<0.0001) with the linear model fitting the data better compared with the second-order polynomial model (ΔAIC = 2) (Fig. 6). For the enriched medium a 2^nd^-order polynomial regression was significant (Table 3, Fig. 6), which is consistent with the predictions of the HSR model and showed a substantially better fit compared with the linear model (ΔAIC = 5.9). Peak estimated egg numbers occurred at 198 larvae/cup (estimated from the regression line, Fig. 6) with observed peak numbers occurring at density range between 143–189 larvae per cup. SPDT is estimated as 395.75 larvae.

**Table 3:** Negative binomial second-order polynomial regression of the effect of larval number on the number of *Ae. albopictus* eggs laid in the enriched medium cups.

	Coefficient	SE	z value	P value
Intercept	5.313	0.133	39.725	**<0.0001**
Larvae	6.711E-3	2.5E-3	2.684	**0.007**
Larvae^2^	-1.650E-05	8.098E-06	–2.037	**0.041**

AIC = 397.12.

### Experiment 3: the effect of conspecific eggs

**Figure 7:**
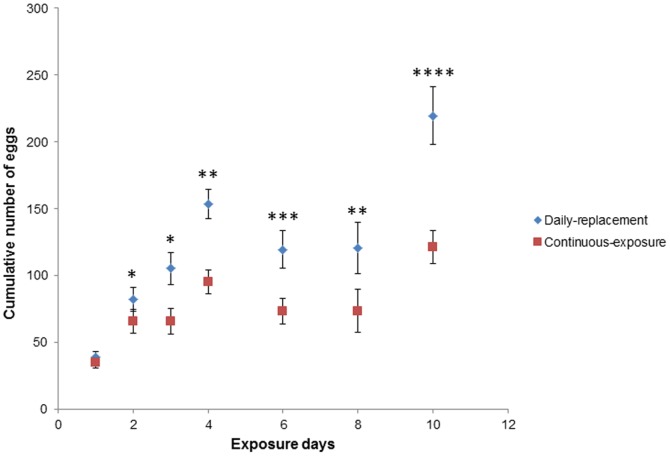
The relationship between oviposition cup exposure time (days) and the mean cumulative number of *Ae. albopictus* eggs (±standard error) laid in these cups for the daily-replacement and the continuous-exposure treatments. (* P <0.05, ** P <0.01, *** P < 0.001, **** P < 0.0001)

As expected, the cumulative number of eggs increased with exposure time (Fig. 7, Table 4). In addition, cumulative number of eggs was higher in the daily-replacement- compared with the continuous-exposure treatment (mean±se: 110.85±6.65 versus 71.01±4.08, respectively) (Table 4, Fig. 7). Yet, as the significant treatment-by-exposure time interaction suggests (Table 4), the effect of the treatment differed over the range of exposure days (Fig. 7). As expected, number of eggs did not differ between the daily replacement and the continuous exposure cups at exposure time of 1 day as both treatments were exposed for the same amount of time. At exposure time of 2 days the difference is small yet significant, and at all subsequent exposure times the difference is large and significant (Fig. 7).

**Table 4:** Negative Binomial Analysis of Deviance table for the effects of treatment (daily-replacement versus continuous exposure), cup exposure time, their interaction, location (Transect), and time (Session) (with the latter two used as control variables) on the cumulative number of eggs laid by *Aedes albopictus* mosquitoes in oviposition cups.

Variable	DF	Deviance	Res. DF	Res. Dev.	P value
NULL			319	594.87	
Treatment	1	34.44	318	556.43	**< 0.0001**
Exposure time	1	116.39	317	440.04	**< 0.0001**
Transect	1	1.27	316	438.77	0.259
Session	6	79.05	310	359.72	**< 0.0001**
Treatment x exposure time	1	5.17	309	354.55	**0.0230**

The results of the control experiment, suggest that this preference for the daily-replacement cups is not due to aversion from aged ovistrips. Number of eggs did not differ between aged and fresh ovistrips for 0, 2, and 5 aging days. However, for ovistrips aged for 10 days number of eggs was actually higher in the aged ovistrips, which is exactly the opposite of what would have been expected due to a confounding effect of aversion from aged ovistrips (Table 5).

**Table 5:** Control experiment.

Paper aging time (days)	No. eggs in aged ovistrip (mean ± SE)	No. eggs in non-aged ovistrip (mean ± SE)	Paired-t test
0	49.87±4.54	52.93± 5.44	t = –0.68, P = 0.50
2	71.95±11.04	73.7410.23	t = –0.14, P = 0.89
5	47.38±4.42	44.56± 6.88	t = 0.51, P = 0.61
10	50.0± 8.15	38.3± 4.72	t = 2.28, P = 0.03

Comparison of the number of eggs laid by *Ae. albopictus* mosquitoes in ovistrips aged in water for varying number of days compared with a non-aged ovistrips.

**Figure 8:**
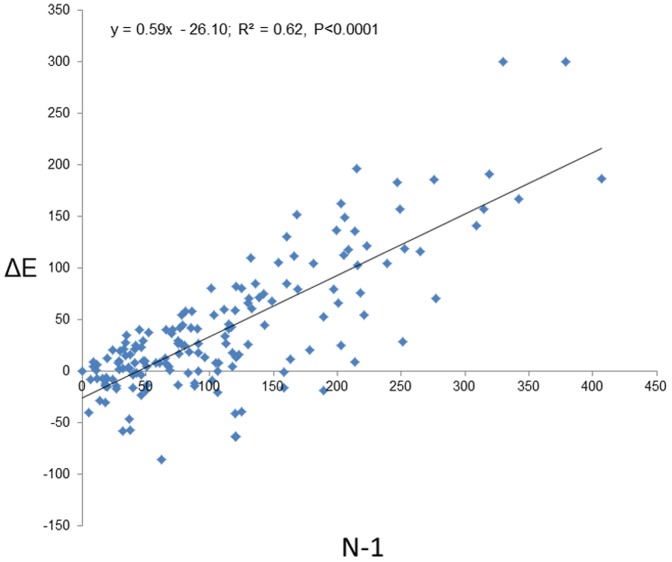
Regression plot of ΔE (the difference between the cumulative number of eggs in the replacement and the exposure cups) against the cumulative number of eggs (minus 1) in the daily-replacement cups.

The regression of ΔE (the difference between the cumulative number of eggs in the replacement and the exposure cups) against the cumulative number of eggs in the daily-replacement cups (minus 1) revealed a line with a significant negative intercept (±se) (–26.10±5.08, t = –5.14, P<0.0001) and positive slope (0.59±0.04, t = 16.31, P<0.0001) (Fig. 8). Such a result is consistent with the predictions of the HSR hypothesis (Fig. 3). The X-axis intercept of 44.24 eggs represents the SPDT below which female mosquitoes prefer to lay eggs where conspecific eggs pre-existed (the conspecific attraction range) and above which they prefer to lay eggs in virgin ovistrips (the conspecific repellence range).

### Meta-analysis

In total, we identified 44 papers addressing the issue of the effect of conspecific immature stages on the oviposition response of mosquitoes (Fig. 4), with a total of n = 91 independent observations (most studies reported on more than one species or more than one experiment) (see Table S1). Nineteen mosquito taxa were reported, with studies on *Cx. quinquefasciatus* being the most common (37%) followed by studies on *Ae. aegypti* (20%), *Ae. triseriatus* (8%), *An. gambiae* (8%), and *Ae. albopictus* (6%). The majority of studies (67%) were conducted in the laboratory and the rest (33%) the field (Table 6). Most studies were conducted on the larval stages (52%), followed by studies using eggs (37%) and pupae (11%) (Table 6). For all the data combined, the proportion of “positive effects” (41.7%) was significantly larger than the expected (proportion test: χ^2^ = 12.75, df = 1, P = 0.0004) and the proportion of “Density-dependent effects” (13.2%) was significantly lower than expected (proportion test: χ^2^ = 6.16, df = 1, P = 0.013). The proportion of “no effects” (25.2%) and “negative effect” (19.8%) did not differ significantly from the 25% expectation. Note, that the number of “No effect” reports (n = 23) might be an under-estimate due to reporting- or publication bias. This distribution of conspecific effects did not differ significantly among mosquito immature stages (contingency table: χ^2^ = 8.84, df = 6, P = 0.18). The distribution of conspecific density effects did differ significantly between lab and field studies (contingency table: χ^2^ = 17.91, df = 3, P = 0.0004). In lab studies (n = 61), the proportion of “positive effects” (52.4%) was significantly larger than expected (proportion test: χ^2^ = 23.08, df = 1, P<0.0001) and the proportion of “negative effects” (8.2%) was significantly smaller than expected (proportion test: χ^2^ = 8.3, df = 1, P = 0.004). In contrast, in field studies (n = 31), the proportion of “negative effects” (43.3%) was significantly larger than expected (proportion test: χ^2^ = 4.44, df = 1, P = 0.035) while the proportion of “positive effects” (23.3%) did not differ from the expected 25% (proportion test: χ^2^ = 0, df = 1, P = 1). In addition, the proportion of “density-dependent effects” differed between lab and field studies with significantly lower proportion (8.2%) than expected (proportion test: χ^2^ = 8.31, df = 1, P = 0.004) in the former and not significantly different (proportion test: χ^2^ = 0.71, df = 1, P = 0.40) in the latter (16.7%). The proportion of “no effects” did not differ significantly from expected for neither study types (30% and 17% for lab and field studies, respectively).

**Table 6:** Summary of a literature review on the effect of conspecific immature stages on the oviposition behavior of gravid mosquitoes based on lab studies (A), Field studies (B), and combined (C).

	No effect	Positive effect	Negative effect	DD effect	N
Laboratory studies					
Eggs	6	10	2	4	22
Larvae	7	17	3	2	29
Pupae	5	4	0	1	10
N	18	31	5	7	61
Field studies					
Eggs	4	3	3	2	12
Larvae	1	4	10	3	18
Pupae	0	0	0	0	0
N	5	7	13	5	30
All studies combined					
Eggs	10	13	5	6	34
Larvae	8	21	13	5	47
Pupae	5	4	0	1	10
N	23	38	18	12	91

See Table S1 for a detailed description of the data.

We evaluated the HSR hypothesis with five mosquito species for which a sufficient amount of data was available on their oviposition response at a range of conspecific immature densities (*Cx. quinquefasciatus*, *Ae. aegypti*, *Ae. triseriatus*, *An. gambiae*, and *Ae. albopictus*). With lab studies on *Cx. quinquefasciatus,* data is congruent with the HSR hypothesis. “Positive effect” was observed at egg raft densities (or egg pheromone concentration equivalents) ranging from 1 all the way to 266 [41], [44]. However, Wachira et al. [36] observed the increasing trend ranging from 0 to 25 egg rafts after which (50 – 100 egg rafts) oviposition rate remained leveled and even slightly decreased. Similarly, Blackwell et al. [57] observed an increasing trend in oviposition rates at egg pheromone ranging from 0.01 to 80 μg, which is equivalent to 0.03 – 266 Egg-rafts. However, a slight increase above that threshold resulted in a sharp drop in this preference. For field studies with *Cx. quinquefasciatus* support for the HSR hypothesis is ambiguous. Consistent with the HSR model, Braks et al. 2007 [58] reported high preference at 1 egg raft but reduced preference at 10 egg rafts, and a “negative effect” was reported by Reisen and Meyer 1990 [31] at 50 egg rafts using CDC traps. However, using the same egg raft number, “no effect” was observed when using an outdoors cage bioassay [31]. In contrast with those observations, field studies using egg pheromone at a single dose equivalent to 16,000 rafts reported a “positive effect”[42], [68]. With *Ae. triseriatus,* oviposition rate was lower in cups where eggs were allowed to accumulate compared with cups in which oviposition substrate were replaced weekly [50]. Yet, in later studies these researchers reported “no effect” [24], [30]. Egg density used was not specified in any of these studies. With *Cx. quinquefasciatus* larvae, no support for the HSR pattern was observed with some studies indicating positive effects over a wide range (0–1 larvae/ml) of larval densities [36] and negative effects at low densities [52].

With *Ae. aegypti larvae*, data were consistent with the HSR hypothesis. Wong *et al.* [34] reported a positive effect of conspecific larvae at low larval density (0.0125 larvae/mL) while Allan and Kline [32] and Zahiri and Rau [60] reported no-effect and a negative effect at intermediate (0.16 larvae/mL) and high (2 larvae/mL) larval density levels, respectively. With *An. gambiae*, McCrae [51] reported a negative effect at larval density higher (1.25 larvae/mL) than its SPDT (0.75–1 larvae/mL) as reported by Sumba [55]. With *Ae. Albopictus*, Allan and Kline [32] reported a positive effect at larval density lower (0.162 larvae/mL) than the SPDT observed in our study (0.30–0.34 larvae/mL, Figures 5, 6).

## Discussion

### The effect of conspecific larvae on mosquito oviposition response

At the larval level, we observed hump-shape relationship, consistent with the HSR model (Fig. 1), for both the no-resource enrichment experiment (experiment 1) (Fig. 5) and the resource-enrichment treatment of experiment 2 (Fig. 6). Results of the no-resource enrichment experiment were highly variable due to, among others, substantial temporal and spatial variability. Nonetheless, after controlling statistically for these sources of error, the hump-shaped pattern proved statistically significant and superior to all other competing models. Results of the resource-enrichment treatment of experiment 2 were less variable but suffered from a relatively small sample size. Nevertheless, here too, the hump-shaped pattern proved statistically significant and superior to all other alternative models. Both experiments used a similar, wide, range of conspecific larval numbers (0–358 and 0–352 larvae, respectively), which enabled the detection of the full range of the expected oviposition responses: an increase in egg deposition with larval density at low-to-intermediate densities and a decrease in egg deposition at intermediate-to-high larval density (Fig. 1). To evaluate whether at high densities the oviposition preference switched from “attraction” to “repellency” we used as a reference the y-intercept of the second order polynomial regression line. Although at intermediate-to-high density range some points fell in the “repellency” range, the grand majority of the data of experiment 1 remained in the “attraction” range. In experiment 2, none of the points at the intermediate-to-high density range fell in the “repellency” range. Furthermore, the expected switching-preference-density-threshold (SPDT) as estimated from their respective regression line equation fell above the range of larval numbers used in these experiments. These results are consistent with the trend observed in our meta-analysis of predominance of “positive effect” (42%) reports. Yet, as demonstrated by the meta-analysis, many of these studies might have missed the hump-shaped relationship by either evaluating oviposition response over a narrow conspecific density range or by using only the low and high ends of this range.

Enrichment of the rearing medium was expected to increase overall oviposition rates and to shift optimal conspecific density and SPDT to higher larval densities due to suppression of the competitive effect (Fig. 1). Indeed, in experiment 2, number of eggs laid was 2.25 higher in the enriched medium compared with the water control. However, due to differential hatching rate that resulted in larger larval-number range in the enriched medium treatment (compared with its control), we could not test the other prediction. Higher hatching rate in enriched medium is a known phenomenon in mosquitoes [69], [70]. Consequentially, in the control medium a linear positive effect was observed whereas in the enriched medium the entire hump-shaped response was observed (Fig. 6). This result is consistent with the HRS model, suggesting that in the un-enriched medium the maximal larvae number was still below the optimum density (N_opt_, Fig. 1) whereas in the enriched medium larval numbers did exceed that threshold. Furthermore, despite the higher larval density in them, higher number of eggs was laid in the enriched medium cups compared with the water control suggesting a stronger effect of resource enrichment compared with the effect of conspecifics, a phenomenon shown previously in *Ae. albopictus* in other studies [24], [30] (but see [5]).

The other three studies reporting a HSR pattern with respect to conspecific larvae were those of Benzon and Apperson [56], Zahiri and Rau [60], and Sumba et al. [55]. Benzon and Apperson ([56] observed an increase followed by a decrease in the preference of *Ae. aegypti* for larval-conditioned water with larval density ranging from 0 to 4 larvae/mL, with peak preference occurring at 2 larvae/mL. Zahiri and Rau [60] used larvae density range of 0.5 to 3 larvae/mL and found hump-shaped relations for 2^nd^ instar *Ae. aegypti* larvae with peak preference also at 2 larvae/mL. For comparison, in our study, peak oviposition activity of *Ae. albopictus* occurred at the densities of 0.30 larvae/mL or 0.34 larvae/mL in the enrichment and the no-enrichment experiments, respectively, suggesting *Ae. albopictus* are more sensitive to intra-specific competition. The only study to date that, successfully, evaluated the role of resources enrichment on the HSR pattern is that of Sumba et al. [55]. Using field enclosures, they found a HSR pattern with *An. gambiae* in an enriched medium. However, in the resource-poor distilled water medium, only a negative effect of conspecific larvae was observed. This was suggested to imply that the production of an attractant larval pheromone could occur only in sites with sufficient resources.

### The effect of conspecific eggs on mosquito oviposition response

At the eggs level, we also found strong support for the hump-shape regulation model. At low egg numbers *Ae. albopictus* females laid more eggs on ovistrips containing pre-existing conspecific eggs. But as the cumulative number of eggs on those ovistrips increased mosquitoes gradually shifted their preference away from them and towards the daily-replacement cups containing virgin ovistrips (Fig. 8). The SPDT (here indicated by the x-axis intercept, Fig. 8) was estimated at 44 eggs per ovistrip (0.1 eggs/mL), which is lower than that estimated in our study for conspecific larvae (0.44 – 0.49 larvae/mL). The negative effect of conspecific eggs probably does not result from competition for oviposition space since at this low-intermediate density range plenty of oviposition space was still available on the water-interface band of the ovistrip (G.W. Personal observation). It is possible however, that at the highest egg density range oviposition space might become limited. Acknowledging the fact that these differences could be attributed to temporal effects (the larvae-effect and the egg-effect experiments were not conducted at the same year), our results suggest that *Ae. albopictus* females are more sensitive to the competitive effect of conspecific eggs than to conspecific larvae. This observation seems counter-intuitive since conspecific eggs probably indicate the potential for future larval competition [33] whereas the presence of conspecific larvae indicates present-time larval competition. However, as Benzon and Apperson (1988) observed, it is possible that this response is mediated via the positive effect of mosquito larvae on the microbial biota through their excrements, which in turn extends the attraction effect of aggregation to higher conspecific larvae densities. The chemical ecology of these interactions remains to be elucidated. For example, in *Cx. quinquefasciatus* erythro-6-acetoxy-5-hexadecanolide from egg apical droplets was identified as a major attractant [27], [44], [68]. For *Ae. albopictus* such an egg-derived pheromone has not yet been identified.

Only four previous studies have reported a HSR pattern with respect to conspecific eggs [36], [57]–[59]. Williams et al. (2008) used single-female bioassays and showed that *Ae. aegypti* had a strong oviposition preference for oviposition substrates containing intermediate numbers of conspecific eggs (11–38 eggs, median: 20) compared with empty- or high-density (39-74 eggs, media: 53) substrates. Interestingly, their estimated SPDT of 53 eggs is close to our estimate of 44 eggs for *Ae. albopictus*. Another study, reported a similar pattern with respect to heterospecific egg [36]. In a laboratory study, Wachira et al. [36] reported hump-shaped relation with gravid *An. gambiae* initially preferring to lay more eggs in cups containing low numbers of *Cx. quinquefasciatus* egg-rafts compared with a no-eggs control, which then switched into avoidance at *Cu. quinquefasciatus* eggs above the density of ca. 16 egg-rafts/100 mL water. In that study, they also evaluated the response of *Cx. quinquefasciatus* to conspecific eggs and found a density-dependent response with oviposition initially increasing up until ca. 20 egg-rafts/100 mL and then leveling-off and slightly decreasing. Similarly, Braks et al. [58] reported a density-dependent effect with positive effect of a single *Cx. quinquefasciatus* egg-raft but no effect or slight decrease at 10 egg-rafts. Finally, Blackwell et al. [57] tested the oviposition response of *Cx. quinquefasciatus* along a gradient of 0.07–80 μg of erythro-6-acetoxy-5-hexadecanolide and found a gradual increase throughout most of this range followed by a sharp drop in preference at 80 μg. The chemical mechanism associated with such density-dependent oviposition preference reduction or switch was shown to be associated with egg semiochemicals. Ganesan et al. (2006) showed with *Ae. aegypti* a concentration-dependent decrease in the attraction of several dodecanoic acids but a concentration-dependent increase in the deterrence/repellence effect with a variety of esters derived from conspecific eggs [65].

### How general is the HSR model?

In this paper, we suggested that the disparate and conflicting results concerning the effect of conspecific immature stages on the oviposition response of mosquitoes could be synthesized by the HSR model with positive-effect observations occurring at the lower range of densities, no-effect occurring at intermediate densities, and negative-effects occurring at high densities. We also suggested that the dearth of observations of the HSR pattern and the conflicting reports regarding these relationships are likely the outcome of the design of most previous studies that tended to explore only a limited range of conspecific densities. To evaluate this hypothesis, we conducted an exhaustive literature review (Fig. 4). From 91 studies, only 12 (13%) reported a density-dependent change in oviposition response consistent with the predictions of the HSR model. It worth mentioning that for the majority of these studies (8 out of 12) (including the larval experiments in this study, Fig. 5, 6) the final oviposition response even at high densities remained at the “attraction” range [27], [36], [56]–[59]. Only four studies [36], [55], [60] (including the conspecific eggs experiment in study, Fig. 8) ended up at the “repellence” range. These “repellence” responses tended to occur either at high conspecific densities or at low resource availability. Consistent with the latter conjecture, is the observation that the SPDT for conspecific eggs in our study was 9–10 times smaller than that estimated for larvae, which might result from the fact that larvae enrich their growth medium with nutrients from their excrements [56] whereas deposited eggs do not. After sorting all studies not reporting HSR and evaluating their oviposition response with respect to the conspecific density in which they were performed, we found a fair amount of support for our expectation that studies reporting “attraction” were conducted at low-intermediate density range whereas “neutrality” and “repellence” occurred at intermediate-to-high conspecific densities. Furthermore, most of the studies reporting “attraction” were conducted either at a narrow range of densities or at the two extremities of the range which might cause them to miss the oviposition peak. On the other hand, many other observations in our analysis were not consistent with this hypothesis or did not have enough information. Hence, the generality of the HSR model in mediating mosquito’s oviposition site-selection needs to be further evaluated. Furthermore, it is important to note that in addition for using a wide density-range of conspecific immature stages, most studies reporting a HSR pattern also used a paired- or multi-choice-design where preference for the conspecific-inhabiting container was evaluated against a biologically-meaningful (often water) control. Given the subtlety of this density-dependent preference switch, we recommend future studies on this topic should continue using such a design.

### Synthesis

Based on the Ideal-Free-Distribution (IFD) theory [71], the oviposition preference-offspring performance (P-P) hypothesis predicts that oviposition-site seeking gravid females should select sites that maximizes the growth and survivorship of their offspring [5], [7]–[9]. So, why do gravid females select, at least at low-to-intermediate densities, to lay eggs at sites containing conspecific immature stages? It has been suggested that the presence conspecific immature stages could indicate the suitability of that site in terms of, among others, food for their larvae, site persistence, lack of predators, and appropriate a-biotic conditions [5], [34], [56], [59]. However, this conjecture has rarely been tested. Specifically, are sites containing conspecific immature stages more suitable than those that do not and do offspring performance, indeed, enhanced in such sites? In a well-designed lab study, Yoshioka et al. [5] demonstrated that gravid *Ae. albopictus* were more attracted to conspecific cues over cues indicating food and selected oviposition sites which were sub-optimal in terms of larval performance. Hence, that study suggests that gravid females might be paying a certain “cost of re-assurance” for their reliance on conspecific cues as a rule-of-thumb [72] indicator of habitat suitability. This would be an important avenue for further exploration. Assessing the fitness-density consequence of mosquito’s oviposition site selection is important as it might have significant theoretical and practical implications for predicting mosquito population dynamics and for mosquito control [10], [21]–[23], [49]. For example, if mosquitoes disperse among oviposition sites in a manner approximating the IFD model [71] then habitat distribution and population dynamics could be predicted based on habitat quality [73]. However, if density-dependent oviposition-site selection incorporates some component of an “Allee-effect” then habitat distribution might not necessarily be consistent with the inherent quality of the oviposition site resulting in an apparently erratic habitat distribution and population dynamics [49], [71]. Hence, failure to fully understand the manner by which oviposition-site selection is regulated might limit our ability to predict and manage mosquitoes and possibly other blood-feeding insect populations.

## Supporting Information

Table S1 **Meta-analysis data-base.**
(DOCX)

Appendix S1 **PRISMA 2009 Checklist.**
(DOC)
