# Supplementary material for: Hump-Shaped Density-Dependent Regulation of Mosquito Oviposition Site-Selection by Conspecific Immature Stages: Theory, Field Test with Aedes albopictus, and a Meta-Analysis
Source: PLoS One. 2014 Mar 28;9(3):e92658. doi: 10.1371/journal.pone.0092658 (PMC3969363; doi:10.1371/journal.pone.0092658)
Supplement: Table S1 — Meta-analysis data-base. (DOCX) [file pone.0092658.s001.docx]

**Table S1. Meta-analysis data-base**. The table details all the studies included in the meta-analysis. Literature search was conducted using ISI-Web of knowledge for all years using search code: Topic=(mosquitoes) AND Topic=(oviposition) AND/OR Topic=(competition) AND/OR Topic =(habitat selection) AND/OR Topic = (conspecific). We supplemented these by relevant studies cited in their respective reference lists. Studies were stratified by immature stage (eggs, larvae, pupae) and study type (Laboratory or field). We sorted these articles according to their finding regarding the effect of immature stage number/density on oviposition rate. For each paper we tried to estimate immature stage number/density based on the information provided in their ‘Methods’ or ‘Results’ sections.

|  | Lab | | | | Field | | | |
| --- | --- | --- | --- | --- | --- | --- | --- | --- |
|  | No effect | Positive | Negative | Density-dependent | No effect | Positive | Negative | Density-dependent |
| Eggs | Allan and Kline 1998 *Ae.albopictus* Eggs #: 161 | Allan and Kline 1998  *Ae. aegypti* Eggs #: 145 | Chadee et al. 1990  *Ae. aegypti*  Eggs #: <50 | Williams et al. 2008  *Ae. aegypti* Eggs #: median:20 | Sumba et al. 2008  *An. gambiae* Eggs #: median:0-30 | Edgerly 1998  *Ae.triseriatus* Eggs #: 0-200 | Kitron et al. 1989  *Ae. triseriatus* Eggs #: unspecified but large | This study: *Ae.albopictus*  Cumulative Eggs #:  Daily renewal: 0-407  Total, continuous exposure: 0-222 |
|  | Dhileepan 1997  Cx. molestus  Eggs #: 10 Egg-rafts |  | Onyabe and Roitberg 1997  *Ae.togoi*  Eggs #: 50 | Wachira et al. 2010  *An. gambiae* versus *Cx.quinquefasciatus*  Eggs #: 0-100 | Chaves et al. 2009. *Cx.quinquefasciatus*  Eggs #: unspecified | Otieno et al. 1988. Cx. quinq. Pheromone dose: 5mg equivalent to 16000 Egg-rafts | Reisen & Meyer 1990. Cx. tarsalis. Egg #: unspecified | Braks et al. 2007. Cx. Quinquefasciatus. Egg-raft #: 1-10 and 3 μg pheromone equivalent to 10 Egg-rafts |
|  | Dhileepan 1997  Cx. Annulirostris  Eggs #: 10 Egg-rafts | Barbosa et al. 2007. *Cx.quinquefasciatus*  Eggs #: 3 egg rafts/250 ml as well as natural and synthetic egg pheromone |  | Wachira et al. 2010  *Cx.quinquefasciatus*  Eggs #: 0-100 rafts | Reisen & Meyer 1990. Cx. quinq. Egg #: ~50 (in outdoors cage) |  | Reisen & Meyer 1990. Cx. quinq. Egg #: ~50  (baited CDC trap) |  |
|  | Ahmadi & McClleland 1983. Ae. sierrensis. Density: Egg holding water (10/ml) | Pavlovich and Rockett 2000  *Ae. aegypti*  Eggs #: 250  *Preference for conspecific vs. heterpspecific eggs |  | Blackwell et al. 1993. Cx. quinquefasciatus. Pheromone dose: 0.01-80μg equivalent to 0.03 – 266 Egg-rafts | Nguyen et al. 2012. *Cx.quinquefasciatus*  Eggs #: unspecified | Mboera et al. 2000. Cx. quinq. Pheromone dose: 5mg equivalent to 16000 Egg-rafts |  |  |
|  | Reisen & Meyer 1990. Cx. Tarsalis. Egg #: unspecified | Pavlovich and Rockett 2000  *Ae. albopictus*  Eggs #: 250  *Preference for conspecific vs. heterpspecific eggs |  |  |  |  |  |  |
|  |  | Osgood 1971 Cx. tarsalis. Egg-raft #:  (1) Egg rearing water. (2) 30 egg rafts (3)Egg pheromone |  |  |  |  |  |  |
|  |  | Bruno & Laurence 1979. Cx. quinq.  Egg-raft #: 4-200. Also showed effect of egg pheromone |  |  |  |  |  |  |
|  |  | Bruno & Laurence 1979. Cx. tarsalis  Egg-raft #: - TBD |  |  |  |  |  |  |
|  |  | Laurence & Pickett 1985. Cx. quinq. Pheromone dose: 0.02μg equivalent to 6.25% of a single egg-raft |  |  |  |  |  |  |
|  | Nakamura 1978. Cx. tritaeniorhynchus . Egg-raft #: 10 | Nakamura 1978. Cx. molestus. Egg-raft #: 10 |  |  |  |  |  |  |
|  |  | Dadd & Kleinjan 1974. Cx. pipiens. Egg #: unspecified |  |  |  |  |  |  |
| Larvae | Allan & Kline 1998  *Ae. aegypti* Density: 0.16/ml | Allan and Kline 1998 *Ae.albopictus* Density: 0.162/ml | McCrae 1984  *An. gambiae*  Density: 1.25/ml | Benzon and Apperson 1988  *Ae. aegypti* Density: 0-4/ml | Blaustein and Kotler 1993 Cu-Longiareolata  Density: 0.01/ml | This study *Ae.albopictus*  Water medium  Density:  0-0.11/ml | Sumba et al. 2008  *An. gambiae* Distilled water  density: 0-2/ml | Sumba et al. 2008  *An. gambiae* Enriched water  Density: 0-2/ml |
|  | Bruno & Laurence 1979. Cx. quinq. Density:0.1-0.4/ml | Onyabe and Roitberg 1997  *Ae.togoi*  Density: >0.33/ml | Munga et al 2006  *An. gambiae*  Density:  0-0.5/ml | Zahiri and Rau 1998. *Ae. aegypti*  Density:  0.5-3/ml |  | Edgerly et al. 1998. late season. *Ae.triseriatus*  Density: 0-0.9/ml | Edgerly et al. 1998. early season. *Ae.triseriatus*  0-0.9/ml | This study  *Ae.albopictus* in enriched medium  Density:  0-0.95/ml |
|  | Reisen & Meyer 1990. Cx. Tarsalis. Density: unspecified | Zahiri et al. 1997  Ae. atropalpus  Density: 0.5/ml | Wachira et al. 2010  *An. gambiae* versus *Cx.quinquefasciatus*  Density:  0-1/ml |  |  | Wong et al. 2011  *Ae. aegypti* Density: 0.0125/ml | Mokany & Shine 2003  Oc. australis  density: 0.02/ml | This study  *Ae.albopictus* in water medium  Density:  0-1.1/ml |
|  | Reisen & Meyer 1990. Cx. quinq. Density: unspecified-larvae conditioned water | Kalpage & Brust 1973 A. atropalpus  Density: un- specified |  |  |  | Heard 1994 Weyomia smithi Density:  20/pitcher | Munga et al 2006  *An. gambiae*  Density:  0-0.5/ml |  |
|  |  | Wachira et al. 2010  *Cx.quinquefasciatus* Density:  0-1/ml |  |  |  |  | Mokany and Shine 2003  *Cx.quinquefasciatus* Density: 0.02/ml |  |
|  |  | Dhileepan 1997  Cx. Annulirostris  Density: 0 - 1/ml |  |  |  |  | Kiflawi et al. 2003 Cu. longiareolata Density: 0.024/ml |  |
|  |  | Dhileepan 1997  Cx. Annulirostris  Density: 0.13/ml |  |  |  |  | Kiflawi et al. 2003 Cx. laticinctus. Density: 0.024/ml |  |
|  | Dhileepan 1997  Cx. molestus  Actual larval density 0.13/ml | Dhileepan 1997  Cx. molestus  Density: larval rearing water 0.13/ml |  |  |  |  | Reiskind & Wilson 2004. *Cx. restuans*  0.016/ml-0.052/ml |  |
|  |  | Bentley et al. 1976. *Ae. triseriatus. Larval holding water.* Density: 1/ml |  |  |  |  | Reisen & Meyer 1990. Cx. tarsalis. Density: unspecified-larvae conditioned water |  |
|  |  | McDaniel et al. 1979. *Ae. triseriatus.* Density: unspecified - larval rearing water |  |  |  |  | Reisen & Meyer 1990. Cx. quinq. Density: unspecified-larvae conditioned water |  |
|  |  | Maire &Langis 1985. Ae. communis. Density: larval holding water |  |  |  |  |  |  |
|  |  | Soman & Reuben 1970. *Ae. aegypti* Density: unspecified |  |  |  |  |  |  |
|  | Ahmadi & McClleland 1983. Ae. sierrensis. Density: larval holding water (1/ml) | Ahmadi & McClleland 1983. Ae. sierrensis. Density: Lab rearing water (mixture of stages) |  |  |  |  |  |  |
|  | Nakamura 1978 Cx. tritaeniorhynchus Density: 0.15/ml | Nakamura 1978. Cx. molestus. Density: 0.15/ml |  |  |  |  |  |  |
|  |  | Tilak et al. 2005. *Ae. aegypti. Density:*  (1) 0.1/ml  (2) larval rearing water |  |  |  |  |  |  |
|  |  | Yoshioka et al. 2012. *Ae. albopictus*  Density: 0-0.27/ml |  |  |  |  |  |  |
|  |  | Dadd & Kleinjan 1974. Cx. pipiens. Density: 1-3/ml |  |  |  |  |  |  |
| Pupae | McCrae 1984  *An. gambiae*  Density: 1.25/ml | Kalpage & Brust 1973 A. atropalpus  Density: un- specified |  | Bruno & Laurence 1979. *Cx.quinq.* Density:0.1-0.4/ml |  |  |  |  |
|  | Ahmadi & McClleland 1983. Ae. sierrensis. Density: larval holding water (1/ml) | Andreadis 1977. Cx. Salinarius. Density: 1/ml (1)pupae (2) pupae holding water |  |  |  |  |  |  |
|  | Reisen & Meyer 1990. Cx. Tarsalis. Density: unspecified | Dadd & Kleinjan 1974. Cx. pipiens. Density: unspecified |  |  |  |  |  |  |
|  | Reisen & Meyer 1990. Cx. quinq. Density: unspecified |  |  |  |  |  |  |  |
|  | Dhileepan 1997  Cx. molestus  Density: 0.13/ml | Dhileepan 1997  Cx. Annulirostris  Density: 0.13/ml |  |  |  |  |  |  |

Acronyms used:

*Ae.: Aedes*

*Cx.: Culex*

*Cu: Culliseta*

*An.: Anopheles*

*Oc. Ochlerotatus*

Literature cited – Appendix 1

1. Ahmadi A, Mcclelland GAH (1983) Oviposition Attractants of the Western Treehole Mosquito, Aedes-Sierrensis. Mosquito News 43: 343-345.

2. Ahmadi A, Mcclelland GAH (1983) Oviposition Attractants of the Western Treehole Mosquito, Aedes-Sierrensis. Mosquito News 43: 343-345.

3. Allan SA, Kline DL (1998) Larval rearing water and preexisting eggs influence oviposition by Aedes aegypti and Ae-albopictus (Diptera : Culicidae). J Med Entomol 35: 943-947.

4. Andreadis TG (1977) Oviposition Attractant of Pupal Origin in Culex-Salinarius. Mosquito News 37: 53-56.

5. Barbosa RMR, Souto A, Eiras AE, Regis L (2007) Laboratory and field evaluation of an oviposition trap for Culex quinquefasciatus (Diptera : Culicidae). Mem Inst Oswaldo Cruz 102: 523-529.

6. Bentley MD, Mcdaniel IN, Lee HP, Stiehl B, Yatagai M (1976) Studies of Aedes-Triseriatus Oviposition Attractants Produced by Larvae of Aedes-Triseriatus and Aedes-Atropalpus (Diptera-Culicidae). J Med Entomol 13: 112-115.

7. Benzon GL, Apperson CS (1988) Reexamination of Chemically Mediated Oviposition Behavior in Aedes-Aegypti (L) (Diptera, Culicidae). J Med Entomol 25: 158-164.

8. Blackwell A, Mordue AJ, Hansson BS, Wadhams LJ, Pickett JA (1993) A Behavioral and Electrophysiological Study of Oviposition Cues for Culex-Quinquefasciatus. Physiological Entomology 18: 343-348.

9. Blaustein L, Kotler BP (1993) Oviposition Habitat Selection by the Mosquito, Culiseta-Longiareolata - Effects of Conspecifics, Food and Green Toad Tadpoles. Ecological Entomology 18: 104-108.

10. Braks MAH, Leal WS, Carde RT (2007) Oviposition responses of gravid female Culex quinquefasciatus to egg rafts and low doses of oviposition pheromone under semifield conditions. Journal of Chemical Ecology 33: 567-578.

11. Bruno DW, Laurence BR (1979) Influence of the Apical Droplet of Culex Egg Rafts on Oviposition of Culex-Pipiens-Fatigans (Diptera, Culicidae). J Med Entomol 16: 300-305.

12. Chadee DD, Corbet PS, Greenwood JJD (1990) Egg-Laying Yellow-Fever Mosquitos Avoid Sites Containing Eggs Laid by Themselves Or by Conspecifics. Entomologia Experimentalis et Applicata 57: 295-298.

13. Chaves LF, Keogh CL, Vazquez-Prokopec GM, Kitron UD (2009) Combined Sewage Overflow Enhances Oviposition of Culex quinquefasciatus (Diptera: Culicidae) in Urban Areas. J Med Entomol 46: 220-226.

14. Dadd RH, Kleinjan JE (1974) Autophagostimulant from Culex-Pipiens Diptera-Culicidae Larvae - Distinction from Other Mosquito Larval Factors. Environmental Entomology 3: 21-28.

15. Dhileepan K (1997) Physical factors and chemical cues in the oviposition behavior of arboviral vectors Culex annulirostris and Culex molestus (Diptera: Culicidae). Environmental Entomology 26: 318-326.

16. Edgerly JS, McFarland M, Morgan P, Livdahl T (1998) A seasonal shift in egg-laying behaviour in response to cues of future competition in a treehole mosquito. Journal of Animal Ecology 67: 805-818.

17. Heard SB (1994) Imperfect Oviposition Decisions by the Pitcher Plant Mosquito (Wyeomyia-Smithii). Evolutionary Ecology 8: 493-502.

18. Kalpage K, Brust R (1973) Oviposition Attractant Produced by Immature *Aedes atropalpus*. Environmental Entomology, 2: 729-730.

19. Kiflawi M, Blaustein L, Mangel M (2003) Oviposition habitat selection by the mosquito *Culiseta longiareolata* in response to risk of predation and conspecific larval density. Ecological Entomology 28: 168-173.

20. Kitron UD, Webb DW, Novak RJ (1989) Oviposition Behavior of Aedes-Triseriatus (Diptera, Culicidae) - Prevalence, Intensity, and Aggregation of Eggs in Oviposition Traps. J Med Entomol 26: 462-467.

21. Laurence BR, Pickett JA (1985) An Oviposition Attractant Pheromone in Culex-Quinquefasciatus Say (Diptera, Culicidae). Bull Entomol Res 75: 283-290.

22. Maire A, Langis R (1985) Oviposition Responses of Aedes (Ochlerotatus)-Communis (Diptera, Culicidae) to Larval Holding Water. J Med Entomol 22: 111-112.

23. Mboera LEG, Takken W, Mdira KY, Pickett JA (2000) Sampling gravid Culex quinquefasciatus (Diptera : Culicidae) in Tanzania with traps baited with synthetic oviposition pheromone and grass infusions. J Med Entomol 37: 172-176.

24. Mccrae AWR (1984) Oviposition by African Malaria Vector Mosquitos .2. Effects of Site Tone, Water Type and Conspecific Immatures on Target Selection by Fresh-Water Anopheles-Gambiae Giles, Sensu-Lato. Ann Trop Med Parasitol 78: 307-318.

25. Mcdaniel IN, Bentley MD, Lee HP, Yatagai M (1979) Studies of Aedes-Triseriatus (Diptera-Culicidae) Oviposition Attractants - Evidence for Attractant Production by Kaolin-Treated Larvae. Canadian Entomologist 111: 143-147.

26. Mokany A, Shine R (2003) Oviposition site selection by mosquitoes is affected by cues from conspecific larvae and anuran tadpoles. Austral Ecology 28: 33-37.

27. Munga S, Minakawa N, Zhou GF, Barrack OAJ, Githeko AK, Yan GY (2006) Effects of larval competitors and predators on oviposition site selection of Anopheles gambiae sensu stricto. J Med Entomol 43: 221-224.

28. Nakamura H (1978) Oviposition preference of *Culex pipiens nwlestus* and C. *tritaeniorhynchus sumnwrosus* onto the waters conditioned by the egg rafts or the larvae. Jpn J Sanit Zool 29: 117-123.

29. Nguyen AT, Williams-Newkirk AJ, Kitron UD, Chaves LF (2012) Seasonal Weather, Nutrients, and Conspecific Presence Impacts on the Southern House Mosquito Oviposition Dynamics in Combined Sewage Overflows. J Med Entomol 49: 1328-1338.

30. Onyabe DY, Roitberg BD (1997) The effect of conspecifics on oviposition site selection and oviposition behaviour in Aedes togoi (Theobold) (Diptera : Culicidae). Canadian Entomologist 129: 1173-1176.

31. Osgood CE (1971) Oviposition Pheromone Associated with Egg Rafts of Culex-Tarsalis - Diptera Culicidae. J Econ Entomol 64: 1038-&.

32. Otieno WA, Onyango TO, Pile MM, Laurence BR, Dawson GW, Wadhams LJ, Pickett JA (1988) A Field Trial of the Synthetic Oviposition Pheromone with Culex-Quinquefasciatus Say (Diptera, Culicidae) in Kenya. Bull Entomol Res 78: 463-478.

33. Pavlovich SG, Rockett CL (2000) Color, bacteria, and mosquito eggs as ovipositional attractants for Aedes aegypti and Aedes albopictus (Diptera : Culicidae). Great Lakes Entomologist 33: 141-153.

34. Reisen WK, Meyer RP (1990) Attractiveness of Selected Oviposition Substrates for Gravid Culex-Tarsalis and Culex-Quinquefasciatus in California. J Am Mosq Control Assoc 6: 244-250.

35. Reiskind MH, Wilson ML (2004) Culex restuans (Diptera : Culicidae) oviposition behavior determined by larval habitat quality and quantity in southeastern Michigan. J Med Entomol 41: 179-186.

36. Soman RS, Reuben R (1970) Studies on Preference Shown by Ovipositing Females of Aedes-Aegypti for Water Containing Immature Stages of Same Species. J Med Entomol 7: 485-&.

37. Sumba LA, Ogbunugafor CB, Deng AL, Hassanali A (2008) Regulation of Oviposition in Anopheles gambiae s.s.: Role of Inter- and Intra-Specific Signals. Journal of Chemical Ecology 34: 1430-1436.

38. Tilak R, Gupta V, Suryam V, Yadav J, Gupta D (2005) A Laboratory Investigation into Oviposition Responses of Aedes aegypti to Some Common Household Substances and Water from Conspecific Larvae. Medical Journal Armed Forces India 61: 227-229.

39. Wachira SW, Ndung'u M, Njagi PGN, Hassanali A (2010) Comparative responses of ovipositing Anopheles gambiae and Culex quinquefasciatus females to the presence of Culex egg rafts and larvae. Med Vet Entomol 24: 369-374.

40. Williams CR, Leach KJ, Wilson NJ, Swart VR (2008) The Allee effect in site choice behaviour of egg-laying dengue vector mosquitoes. Tropical Biomedicine 25: 140-144.

41. Wong J, Stoddard ST, Astete H, Morrison AC, Scott TW (2011) Oviposition Site Selection by the Dengue Vector Aedes aegypti and Its Implications for Dengue Control. Plos Neglected Tropical Diseases 5: 1-12.

42. Yoshioka M, Couret J, Kim F, McMillan J, Burkot TR, Dotson EM, Kitron U, Vazquez-Prokopec GM (2012) Diet and density dependent competition affect larval performance and oviposition site selection in the mosquito species Aedes albopictus (Diptera: Culicidae). Parasites & Vectors 5.

43. Zahiri N, Rau ME (1998) Oviposition attraction and repellency of Aedes aegypti (Diptera : Culicidae) to waters from conspecific larvae subjected to crowding, confinement, starvation, or infection. J Med Entomol 35: 782-787.

44. Zahiri N, Rau ME, Lewis DJ (1997) Oviposition responses of Aedes aegypti and Ae-atropalpus (Diptera: Culicidae) females to waters from conspecific and heterospecific normal larvae and from larvae infected with Plagiorchis elegans (Trematoda: Plagiorchiidae). J Med Entomol 34: 565-568.
